# Nanoscale dynamics and localization of single endogenous mRNAs in stress granules

**DOI:** 10.1093/nar/gkae588

**Published:** 2024-07-29

**Authors:** Ko Sugawara, Shin-nosuke Uno, Mako Kamiya, Akihiko Sakamoto, Yasuteru Urano, Takashi Funatsu, Kohki Okabe

**Affiliations:** Graduate School of Pharmaceutical Sciences, The University of Tokyo, Tokyo 113-0033, Japan; RIKEN Center for Biosystems Dynamics Research, Hyogo 650-0047, Japan; Graduate School of Medicine, The University of Tokyo, Tokyo 113-0033, Japan; Graduate School of Medicine, The University of Tokyo, Tokyo 113-0033, Japan; Department of Life Science and Technology, Tokyo Institute of Technology, Kanagawa 226-8501, Japan; Graduate School of Pharmaceutical Sciences, The University of Tokyo, Tokyo 113-0033, Japan; Department of Pharmacology, Yamaguchi University Graduate School of Medicine, Yamaguchi 755-8505, Japan; Graduate School of Pharmaceutical Sciences, The University of Tokyo, Tokyo 113-0033, Japan; Graduate School of Medicine, The University of Tokyo, Tokyo 113-0033, Japan; Graduate School of Pharmaceutical Sciences, The University of Tokyo, Tokyo 113-0033, Japan; Graduate School of Pharmaceutical Sciences, The University of Tokyo, Tokyo 113-0033, Japan; JST, PRESTO, Saitama 332-0012, Japan

## Abstract

Stress granules (SGs) are cytoplasmic messenger ribonucleoprotein granules transiently formed in stressed mammalian cells. Although SG components have been well characterized, detailed insights into the molecular behavior inside SGs remain unresolved. We investigated nanoscale dynamics and localization of endogenous mRNAs in SGs combining single mRNA tracking and super-resolution localization microscopy. First, we developed a methodology for tracking single mRNAs within SGs, revealing that although mRNAs in SGs are mainly stationary (∼40%), they also move in a confined (∼25%) or freely diffusing (∼35%) manner. Second, the super-resolution localization microscopy showed that the mRNAs in SGs are heterogeneously distributed and partially form high-density clusters. Third, we simultaneously performed single mRNA tracking and super-resolution microscopy in SGs, demonstrating that single mRNA trajectories are mainly found around high-density clusters. Finally, a quantitative analysis of mRNA localization and dynamics during stress removal was conducted using live super-resolution imaging and single-molecule tracking. These results suggest that SGs have a highly organized structure that enables dynamic regulation of the mRNAs at the nanoscale, which is responsible for the ordered formation and the wide variety of functions of SGs.

## Graphical Abstract

**Graphical Abstract:**
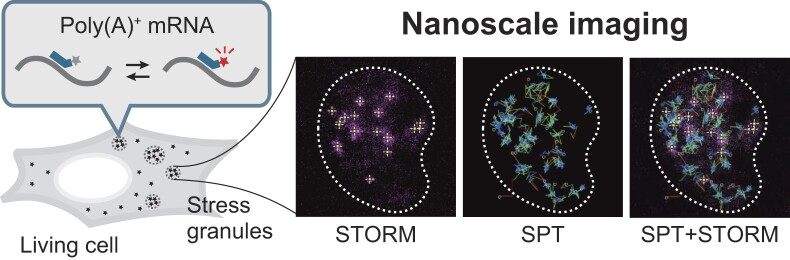

## Introduction

Recent studies indicate that RNA granules play critical roles in post-transcriptional regulation (1–3). In particular, stress granules (SGs) are functional RNA granules dynamically formed in response to environmental stresses such as heat shock or oxidative stress (4). Previous studies revealed that SGs contain a wide variety of molecules such as mRNAs, RNA binding proteins and translation initiation factors (5,6). Despite intense studies on the components of SGs (6,7), the underlying mechanisms of how mRNAs are regulated inside SGs have remained elusive.

Recently, several studies using super-resolution microscopy showed that SG components form stable cores surrounded by liquid-like shell regions (6,8–12). The hypothesis to arise from these studies is that mRNAs assemble into stable cores first by RNA–RNA interactions and protein-protein interactions (13), and then the cores move closer to each other via weak interactions such as liquid–liquid phase separations (LLPS) (6,9,10,12). As noted above, super-resolution microscopy has shed light on the nanoscale structure of SGs by resolving the detailed distribution of SG components in fixed cells. Because SGs assemble and disassemble in an organized manner in response to stress, it is important to understand the dynamics of the components in SGs and their localization. In particular, the heterogeneous distribution of the components in SGs might influence the dynamics of the molecules. This has been raised in a recent review (9), where it has become the subject of interest as to whether SG components in the core layer are less dynamic when compared with those in the shell structure (9,11). Thus, understanding the dynamic states of mRNA molecules, as a key component of SGs, within the newly discovered framework of a core and shell model should further our understanding of the mechanisms of SG formation.

Investigating the dynamics and flux of mRNA within SGs is crucial for understanding the reversible formation and dissociation of SGs. In previous studies, fluorescence recovery after photobleaching (FRAP) was used to analyze the dynamics of components in SGs (14,15). These experiments showed that SG components have fast- and/or slow-recovering fractions with an additional fraction immobile, indicating that SGs contain molecules with various mobilities. Recent studies have employed single molecule tracking to investigate the behavior of individual SG components at the single molecule resolution, targeting RNA-binding proteins (11) and mRNAs (16,17). However, it remains challenging to apply this technique to simultaneously investigate various types of highly expressed mRNAs inside SGs because conventional mRNA labelling methods are not suitable for the densely-packed environment of SGs. Most existing mRNA imaging techniques label the target mRNAs with a relatively high concentration of fluorescent probes and visualize them at the same time (18–23), thereby burying single mRNA signals within densely-packed structures.

To overcome this limitation, we here used the blinking properties of synthetic fluorophores (24–28) to label mRNA by conjugating them to antisense 2′-*O*-methyl RNA probe (14,22). By activating only a small subset of probes in each frame, we were able to detect and track single endogenous mRNA molecules in SGs. By using this blinking probe, we investigated nanoscale dynamics and localization of mRNAs in SGs at the single molecule level. First, we developed a method for visualizing and tracking individual mRNA molecules within densely packed SGs based on the characteristics of a blinking fluorophore. Second, to determine the structural basis that organizes single mRNA behavior, we have examined nanoscale localization of mRNAs inside SGs by super-resolution localization microscopy. Third, we combined single mRNA tracking with super-resolution localization microscopy to associate nanoscale dynamics with nanoscale localization. Finally, the localization and dynamics of mRNA during stress removal were quantitatively analyzed using live super-resolution imaging and single-molecule tracking. In this study, we report that SGs are highly-organized structures and contain nanoscale regions that can tether mRNA molecules, which would be responsible for the formation of nanoscale clusters working as a basic structural unit of large SGs.

## Materials and methods

### Preparation of fluorescently labeled 2′-*O*-methyl RNA probes

The Cy5-labeled poly(U)_22_ 2′-*O*-methyl RNA probe was purchased from FASMAC (Atsugi, Japan), which contains Cy5 at its 5′ end and biotin at its 3′ end (5′-Cy5-UUUUUUUUUUUUUUUUUUUUUU–biotin-3′). The amine-modified poly(U)_22_ 2′-*O*-methyl RNAs (FASMAC, Atsugi, Japan) were reacted with *N*-hydroxysulfosuccinimide (NHS) esters of either ATTO647N (ATTO-TEC, Eichenhang, Germany) or 2MeSiR (15) for the preparation of the ATTO647N-labeled poly(U)_22_ 2′-*O*-methyl RNA probe targeting the poly(A) tail of endogenous mRNA (5′-ATTO647N-UUUUUUUUUUUUUUUUUUUUUU-biotin-3′) and the 2MeSiR-labeled poly(U)_22_ 2′-*O*-methyl RNA probe (5′-2MeSiR-UUUUUUUUUUUUUUUUUUUUUU-biotin-3′), respectively, with both targeting the poly(A) tail of endogenous mRNA. Streptavidin was then attached to the 2′-*O*-methyl RNA probes via biotin at its 3′ end to prevent the accumulation of probes in the nucleus, as reported previously (14). The concentration of each probe was determined using a UV-visible spectrophotometer (V- 570; JASCO, Hachioji, Japan) and adjusted for each experiment.

### Sample preparation for the single-molecule blinking assay on the coverslip

Evaluation of single-molecule blinking was performed using the poly(U)_22_ 2′-*O*-methyl RNA probe conjugated with fluorophores at its 5′ end and streptavidin at its 3′ end via biotin. Prior to sample preparation, coverslips (number 1.5; Matsunami, Kishiwada, Japan) were cleaned with Femto plasma cleaner (Diener electronic, Ebhausen, Germany). Pre-cleaned coverslips were coated with biotinylated bovine serum albumin (BSA), followed by three washing steps with phosphate buffered saline (PBS) containing 137 mM NaCl, 2.68 mM KCl, 8.10 mM Na_2_HPO_4_ and 1.47 mM KH_2_PO_4_ (pH 7.4). Fifty nanomolar fluorescently labeled poly(U)_22_ 2′-*O*-methyl RNA probes were attached to the glass surface via the biotin-streptavidin interaction, allowing dyes to immobilized in a sparse manner to avoid disturbing signals from other emitters. Subsequently, coverslips were washed with PBS three times to remove unbound probes. Finally, coverslips were placed onto slides mounted with the imaging solution (PBS (pH 7.4) containing 14.3 mM 2-mercaptoethanol (βME)) and sealed with clear nail polish. PBS with or without βME was used to investigate the influence of βME on 2MeSiR blinking (Supplementary Figure S1).

### Single-molecule fluorescence imaging on the coverslip

Single-molecule fluorescence imaging of antisense probes on the coverslip was carried out using an inverted microscope system (N-STORM; Nikon, Tokyo, Japan). *In vitro* evaluation of the blinking properties of Cy5, ATTO647N and 2MeSiR by total internal reflection fluorescence microscopy with a 647-nm (∼17.5 W/cm^2^) laser was performed. For each fluorophore, several series of images were acquired at a frame rate of 55 Hz. Images were recorded continuously for 10 000 frames. During observation, the focus was automatically adjusted using the focus stabilization unit (Perfect Focus System; Nikon).

### Single-molecule blinking analysis

Single-molecule blinking properties were analyzed using self-developed software that was designed as a plugin for ImageJ (29,49). Briefly, the positions of fluorophores were detected from a maximum intensity projection of a series of images, followed by a tracing time course of fluorescence intensity of each spot. The fluorophores that emitted more photons than the threshold values were considered to be in a bright state and other fluorophores were considered to be in a dark state or a permanently bleached state. Threshold values were calculated automatically from the base line with a signal-to-noise ratio of five. Based on this classification, fluorescence-on duration, fluorescence-off duration, the number of on events and the number of photons per frame were calculated. These parameters were statistically analyzed for comparison.

### Cell culturing

COS7 cells (Riken, Tsukuba, Japan) were incubated in DMEM (Thermo Fisher Scientific, Waltham, USA) with 10% fetal bovine serum (Thermo Fisher Scientific) containing 50 U/ml penicillin, 50 μg/ml streptomycin (Thermo Fisher Scientific), 2 mM l-glutamine (Thermo Fisher Scientific), 1 mM MEM sodium pyruvate (Thermo Fisher Scientific) and 0.1 mM MEM non-essential amino acids (Thermo Fisher Scientific) at 37°C in 5% CO_2_. For live-cell imaging, cells were cultured in 35 mm glass bottom dishes with grids (AGC Techno Glass, Haibara, Japan) and just prior to the experiment, the medium was replaced with phenol red-free DMEM containing HEPES buffer (Thermo Fisher Scientific).

### Microinjection of the probe

Microinjection of a fluorescently labeled 2′-*O*-methyl RNA probe was performed with a Femtojet Microinjector (Eppendorf, Hamburg, Germany) combined with a micromanipulator (Eppendorf). Prior to microinjection, the probe was diluted in the microinjection buffer containing 80 mM KCl, 10 mM K_2_PO_4_ and 4 mM NaCl pH 7.2, followed by filtering with an Ultrafree-MC filter (Millipore, Billerica, MA, USA). The concentration of the probes was adjusted to 300–1000 nM for single mRNA tracking imaging and 10 μM for super-resolution localization microscopy. The final concentrations of probes in cells were estimated to be 10–30 nM and 300 nM respectively, with a bound ratio of 30–50%.

### Induction and removal of stress

In the process of stress granule formation, sodium arsenite solution (Sigma-Aldrich, St. Louis, USA) was added to DMEM to a final concentration of 0.5 mM. The stress was removed by replacing the solution with one that did not contain sodium arsenite.

### Emetine treatment

After replacing the medium with DMEM containing 10 μg/ml Emetine, the fluorescently labeled poly(U)_22_ 2′-*O*-methyl RNA probe was microinjected. Arsenite stress was induced 30 min after the introduction of Emetine.

### Single-molecule fluorescence imaging in living cells

Single-molecule fluorescence imaging was performed by using N-STORM. For live cell imaging, HILO microscopy with a continuous 647-nm (35 W/cm^2^) laser was employed. For each experiment, 20 000–30 000 frames of sequential images were acquired at a frame rate of 55 Hz. The focus was adjusted using the Perfect Focus System.

### Sample preparation for super-resolution localization microscopy in fixed COS7 cells

Ten micromolar of the Cy5 labeled 2′-*O*-methyl RNA probe was microinjected into COS7 cells on 35 mm glass bottom dishes as described above. At 10 or 45 min after the addition of the arsenite solution, cells were fixed with 3% paraformaldehyde and 0.1% glutaraldehyde. To enhance the blinking performance of Cy5, a 0.1% NaBH_4_ solution was reacted for 7 min and washed with 0.02% TritonX-100 and PBS. Fluorescent beads (FluoSpheresTM Carboxylate-Modified Microspheres, 0.1 μm, red fluorescent (580/605), 2% solids; Thermo Fisher Scientific, Waltham, USA) were placed onto the glass with 2000-fold dilution and washed with PBS after 1 min. The microwells on the glass bottom dishes were filled with the solution (Tris–HCl buffer (pH 8.0) containing 2.25 mg/ml glucose, 25 U/ml glucose oxidase, 25 U/ml catalase and 71.5 mM of βME, and subsequently covered with 18 × 18 mm coverslips (number 1; Matsunami, Kishiwada, Japan).

### Super-resolution localization microscopy in fixed COS7 cells

Super-resolution localization microscopy was performed by using N-STORM. Cy5-labeled antisense 2′-*O*-methyl RNA probes were microinjected into COS7 cells and 0.5 mM sodium arsenite was added to induce SGs in the cytoplasm. We performed imaging in the presence of thiols (71.5 mM βME) and with an oxygen scavenger system to ensure effective Cy5 blinking. Furthermore, to achieve better resolution by enhancing blinking performance, samples were treated with sodium borohydride (NaBH_4_) as described in a previous study (30). Imaging was performed by HILO microscopy with a continuous 647-nm (700 W/cm^2^) laser employed. For each experiment, 100 000 frames of sequential images were acquired at a frame rate of 55 Hz and the first 60 000 frames were used for further analysis. During image acquisition, the focus was adjusted using the Perfect Focus System. Image analysis for localization microscopy was performed using the N-STORM analysis software. The signals from fluorescent beads were used for confirmation of the drift correction. The positions of SGs were determined based on the reconstructed images with a threshold size of 0.3 μm^2^. Cores were extracted by the DBSCAN algorithm, where 25 nm was used for epsilon (the distance for clustering neighboring points) and 100 was used for minPts (the number of neighboring points for cluster points).

### Single mRNA tracking and super-resolution localization microscopy in living COS7 cells

Simultaneous acquisition of single mRNA tracking and super-resolution localization microscopy was performed by using N-STORM. We employed two fluorescently labeled antisense 2′-*O*-methyl RNA probes targeting poly(A)^+^ mRNAs; one probe was labeled with HMSiR (31), which has suitable blinking properties for super-resolution localization microscopy, and the other probe was labeled with Cy3B, which shows good tracking performance among tested green laser-excitable fluorophores. Imaging was performed by HILO microscopy with alternate excitation using 561-nm (for Cy3B; 52.5 W/cm^2^) and 647-nm (for HMSiR; 70 W/cm^2^) lasers. For each experiment, 20 000 time points of sequential images with red and green channels were acquired at a frame rate of 27.5 Hz for each step, and the last 10 000 time points were used for further analysis. During image acquisition, the focus was adjusted using the Perfect Focus System. For the 647-nm excitation channel, image analysis for localization microscopy was performed using the ThunderSTORM ImageJ plugin (32). For the 561-nm excitation channel, single-molecule signals were detected and localized using the ThunderSTORM ImageJ plugin (32), followed by single particle tracking with TrackMate (33). For making trajectories, the nearest neighbor algorithm was used and the maximum travel distance was set to 400 nm. Trajectories over 720 ms (20 points) were extracted for further analysis. The positions of SGs were determined based on the reconstructed images with a threshold size of 0.3 μm^2^. Cores were extracted by the DBSCAN algorithm, where 25 nm was used for epsilon and 40 was used for minPts. For the live super-resolution imaging shown in Figure 5A and Supplementary Video 2, Cy3B-labeled antisense 2′-*O*-methyl RNA probes were microinjected into COS7 cells, followed by adding and removing stress as described above. In this experiment, the medium was phenol red-free DMEM containing HEPES buffer, 2.25 mg/ml glucose, 25 U/ml glucose oxidase, 25 U/ml catalase, and 71.5 mM of βME. Imaging was performed by HILO microscopy with a continuous 561-nm (52.5 W/cm^2^) laser, and 200 000 frames of sequential images were acquired at a frame rate of 55 Hz. During image acquisition, the focus was adjusted using the Perfect Focus System. Image analysis for localization microscopy was performed using the N-STORM analysis software. The super-resolution image sequence was reconstructed using QuickPALM (34), with 5000 neighbor frames accumulated every 1000 frames.

### Radial distribution function analysis for SPT-STORM imaging

For each SGs region, the distances between tracking points and its nearest cores were calculated. The radial distribution function of the distances was calculated with a bin size of 10 nm and then normalized with the value at 500 nm. The radial distribution function calculated for the randomly distributed points and its nearest cores was used to generate the relative radial distribution function shown in Figure 4D.

### Single-molecule detection and single particle tracking

Single-molecule signals were detected and localized using the ThunderSTORM ImageJ plugin (32), followed by single particle tracking with TrackMate (33). For making trajectories from detected points, the nearest neighbor algorithm was used and the maximum travel distance between any two points in consecutive frames was set to 400 nm. Trajectories over 720 ms (40 points) were extracted for further analysis. The distributions of track durations are shown in Supplementary Figure S3.

### MSD analysis and categorization of tracks

MSD analysis and categorization of tracks were performed in a similar way as reported previously (35,36). MSD for each trajectory was calculated by Equation (1):


(1) \begin{equation*}{\mathrm{MSD}}\left( {{\mathrm{t}} = {\mathrm{n}} \cdot \Delta {\mathrm{t}}} \right) = {\mathrm{\;}}\frac{{\mathop \sum \nolimits_{i = 1}^{N - n} \left[ {{{\left( {{x_{i + n}} - {x_i}} \right)}^2} + {{\left( {{y_{i + n}} - {y_i}} \right)}^2}} \right]}}{{N - n}}\end{equation*}

where *N* is the time window for analysis (40 frames for the results in Figure 2), *n* is the frame number to be analyzed, Δ*t* is the time resolution (18 ms for the results in Figure 2), and *x*_*i*_ and *y*_*i*_ are the coordinates of the detected points at frame *i*. Categorization of tracks was performed as follows. First, the trajectories were sorted into either the stationary or mobile mode based on the initial slope of the MSD (between 18 and 180 ms) fitted by linear regression. Trajectories that have smaller slopes than the threshold value (*D*_threshold_) were categorized as the stationary mode and the remaining trajectories were grouped into the mobile mode; the threshold value was calculated based on the spatial resolution (∼0.05 μm): *D*_threshold_ = (0.05 μm)^2^/(4 × 9 × 0.018 s) ∼ 0.004 μm^2^ s^–1^. Subsequently, for the mobile mode, each MSD between 18 and 396 ms was fitted by Equation (2):


(2) \begin{equation*}{\mathrm{MSD}}\left( {\mathrm{t}} \right){\mathrm{\;}} = {\mathrm{\;}}\frac{{4{\mathrm{R}}_{{\mathrm{conf}}}^2}}{3}\left( {1 - {e^{ - t/{\mathrm{\tau }}}}} \right)\end{equation*}

where *R*_conf_ is the confinement radius and *τ* is the time constant defined as *τ* = (*R*^2^_conf_/3*D*_conf_). Trajectories with smaller *τ* than half of the time interval for curve fitting (198 ms) were categorized as the confined mode and the remaining trajectories were grouped into the diffusive mode. In the diffusive mode, *D*_diff_ was calculated from the slope of the MSD (between 18 and 396 ms) fitted by linear regression, as shown in Equation (3).


(3) \begin{equation*}{\mathrm{MSD}}\left( {\mathrm{t}} \right){\mathrm{\;}} = 4{{\mathrm{D}}_{{\mathrm{diff}}}}\;t\end{equation*}

After categorization, the mean value and standard error of the mean were calculated for each category to draw MSD-t plots. Statistical tests for calculated variables were performed by using the Kruskal–Wallis test followed by the Steel–Dwass–Critchlow–Fligner post hoc test.

### Analysis of influx and efflux of mRNA in SGs

Each mRNA tracking result was classified into influx and efflux categories based on whether the first and last frames were inside or outside the SGs region. A classification based on diffusion modes was also included and summarized in the table. Both absolute numbers and ratios were compared for analysis.

## Results

### Development of a blinking-based single mRNA tracking approach

**Figure 1.**
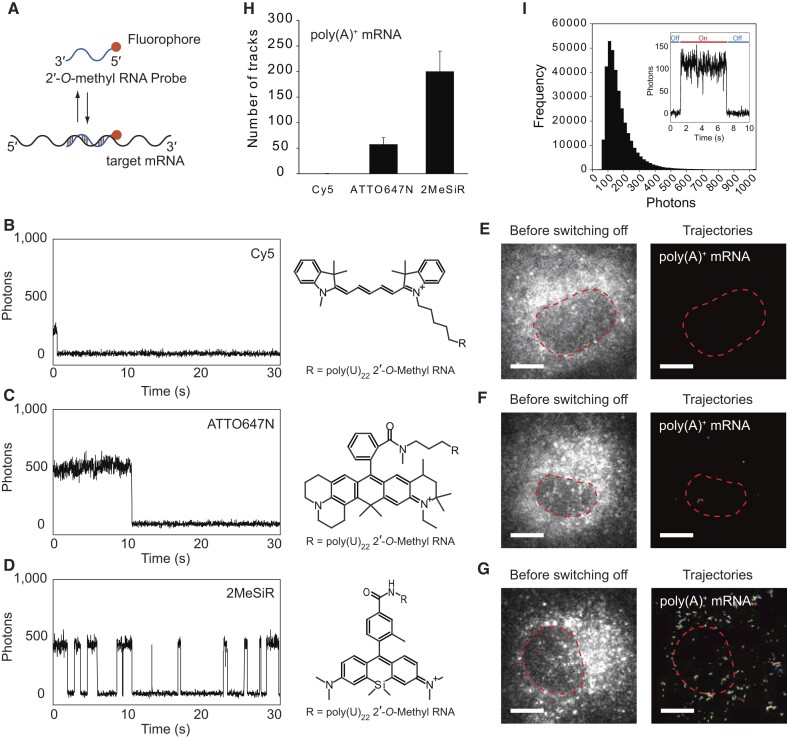
Blinking-based single mRNA tracking methodology. (**A**) Schematic of the fluorescently labeled 2′-*O*-methyl RNA oligonucleotide binding to a target mRNA. (B–D) Exemplary single-molecule fluorescence time traces for (**B**) Cy5, (**C**) ATTO647N and (**D**) 2MeSiR. Poly(U)_22_ 2′-*O*-methyl RNA-bound fluorophores were attached to the coverslip and the measurements were performed in the presence of 14.3 mM βME. The fluorescence time courses were recorded just after starting excitation. (E–G) Exemplary fluorescence images of poly(A)^+^ mRNA in living COS7 cells before switching off (left) and trajectories (right). Poly(A)^+^ mRNA molecules were visualized with 2′-*O*-methyl RNA oligonucleotides labeled with (**E**) Cy5, (**F**) ATTO647N and (**G**) 2MeSiR. The red broken lines on the images indicate the positions of the nuclei in cells. The trajectories were arbitrary colored to make them distinguishable. (**H**) Detected number of tracks per cell using Cy5, ATTO647N or 2MeSiR-labeled poly(U)_22_ 2′-*O*-methyl RNA probes in living COS7 cells measured between 5001 frames and 15 000 frames after starting excitation (*n* = 9 cells for each condition). (**I**) Histogram of the number of photons emitted from fluorescent particles. The inset shows an exemplary time course of fluorescence intensity of a particle. Scale bars, 10 μm (**E**–**G**).

A method for single mRNA tracking suitable for use in a dense environment such as SGs was developed. The basic concept is similar to the sptPALM technique (27), which labels target proteins with photoactivatable fluorescent proteins that activate only a small subset of fluorescent proteins in single frames to visualize single individual molecules in the dense environment. We implemented this concept by labelling oligonucleotide-based probes with synthetic fluorophores to achieve single mRNA imaging in SGs. We evaluated the optimal fluorophore for blinking-based single mRNA tracking. To minimize the influence of autofluorescence during observations, we focused on red laser-excitable fluorophores and selected three dyes with different types of structures: Cy5 (a cyanine dye), ATTO647N (a carbopyronine dye) and 2MeSiR (a silicon rhodamine dye) (31). We first measured and evaluated four photophysical parameters in vitro to characterize the blinking properties of the fluorophores: (i) number of blinking events; (ii) fluorescence- on duration per event; (iii) fluorescence-off duration per event and (iv) number of photons per frame. Fluorescently labeled poly(U)_22_ 2′-*O*-methyl RNA probes (Figure 1A) were attached to a coverslip to analyze the blinking properties of the fluorophores. The evaluation was performed in a buffer at physiological pH that contained millimolar levels of thiols, which corresponds to intracellular glutathione levels (37). No other reagents, e.g. an oxygen scavenger system, were added. Although both Cy5 and ATTO647N are reported to show blinking performances in the presence of the oxygen scavenger system (38), they reverted to the stable dark state or photobleaching within several frames under our experimental conditions (Figure 1B, C), indicating that they are not suitable for recursive tracking of single molecules in the presence of oxygen. In contrast to Cy5 and ATTO647N, 2MeSiR showed reversible blinking in the same buffer conditions (Figure 1D). In addition, the on duration (1.0 s) and photons per frame (268 photons) were favorable for single-particle tracking (Supplementary Table S1). In the absence of βME, blinking of 2MeSiR was not observed (Supplementary Figure S1), confirming that thiols are crucial for the blinking performance of 2MeSiR. We postulate that the relatively stable dark state of 2MeSiR is a radical anion or a reduced form based on a previous report (26).

Following the *in vitro* evaluation, blinking properties of the fluorophores were tested in living cells. We microinjected fluorescently labeled poly(U)_22_ 2′-*O-*methyl RNA probes into living COS7 cells targeting the poly(A) tail of endogenous mRNAs. After several tens of minutes, allowing the probes to bind to mRNAs, images were acquired using highly inclined and laminated optical sheet (HILO) microscopy (39) in Dulbecco's modified Eagle's medium (DMEM) containing 4-(2-hydroxyethyl)-1-piperazineethanesulfonic acid (HEPES) buffer without additional thiols or oxygen scavengers, utilizing intracellular glutathione as a possible blinking inducer. We measured the number of tracks between 5000 (∼90 s) and 15 000 frames (∼270 s) after initiating excitation, during which most of the dyes moved into the dark state or photobleaching from the initial bright state. Among the three dyes examined, 2MeSiR produced the highest number of trackable signals, whereas the majority of Cy5 and ATTO647N moved into the stable dark state or photobleaching within several tens of seconds, providing few or no trajectories (Figure 1E–H). These results were in accord with those of the *in vitro* assay and indicated that intracellular thiol levels are suitable for the blinking performance of 2MeSiR.

To confirm that we can actually visualize single individual mRNA molecules in living cells, we calculated the number of photons that single particles emitted in each trajectory and analyzed their time trace. The histogram showed a single peak and the time course of fluorescence intensity represented a single-step decrease (Figure 1I). These results indicate that the analyzed particles represent single mRNA molecules.

Based on our experimental results, we concluded that 2MeSiR is a suitable fluorophore for blinking-based single mRNA tracking.

### Observation of nanoscale behavior of single poly(A)^+^ mRNA molecules in SGs

**Figure 2.**
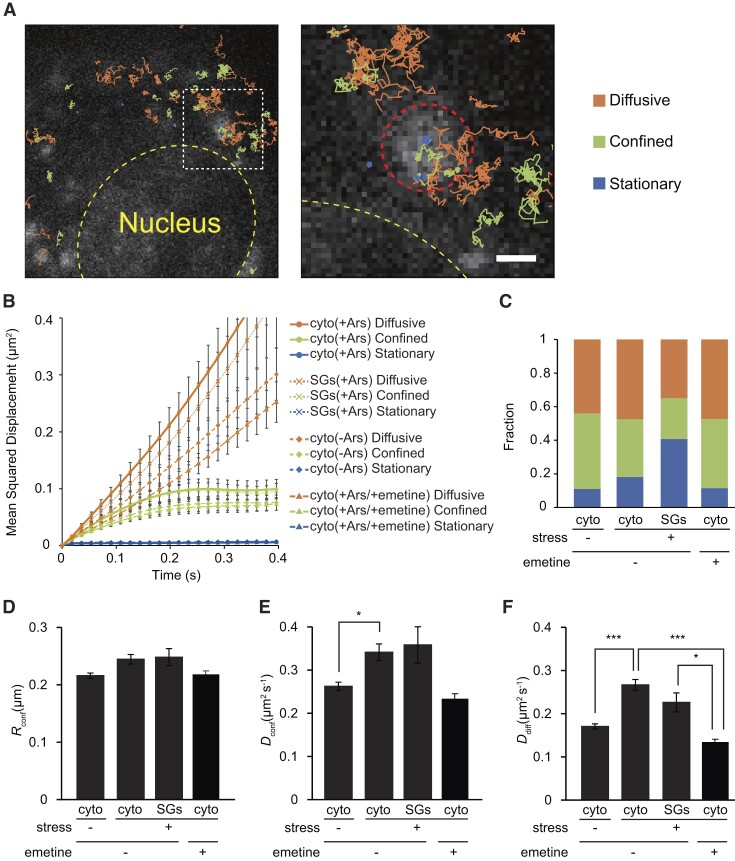
Tracking of single endogenous poly(A)^+^ mRNA in SGs in living COS7 cells. (**A**) Exemplary trajectories of single endogenous poly(A)^+^ mRNAs in a stressed cell. Trajectories are overlaid on the epi-fluorescence image of poly(A)^+^ mRNAs with color-coding based on the diffusion modes: diffusive (orange), confined (green) and stationary (blue). Scale bar, 5 μm. The boxed area in the left image indicated with white broken lines is shown at a higher magnification in the right image; scale bar, 1 μm. (**B**) Mean squared displacement (MSD) as a function of time for poly(A)^+^ mRNA in the cytoplasm (cyto) and in SGs under stressed (+Ars), unstressed (–Ars), or stressed with emetine treatment (+Ars/+emetine) conditions, as indicated on the right. (mean ± s.e.m. for trajectories; *n* ≥ 76 trajectories from nine cells; *n* ≥ 68 trajectories from three cells (+Ars/+emetine)). (**C**) Fraction of diffusive, confined, and stationary modes in the cytoplasm (cyto) in normal cells (cyto; stress (–); emetine (–)), in the cytoplasm (cyto; stress (+); emetine (–)) or SGs (SGs; stress (+); emetine (–)) in stressed cells, and in the cytoplasm in stressed cells with emetine treatment (cyto; stress (+); emetine (+)) (mean for cells; *n* ≥ 3 cells). (**D**) Radii of confinement calculated for each condition (mean ± s.e.m. for trajectories; *n* ≥ 76 trajectories from ≥3 cells). (**E**) Diffusion coefficients for confinement mode calculated for each condition (mean ± s.e.m. for trajectories; *n* ≥ 76 trajectories from ≥3 cells; *P ≤ 0.05). (**F**) Diffusion coefficients for diffusion mode calculated for each condition (mean ± s.e.m. for trajectories; n ≥ 109 trajectories from ≥ 3 cells; *P ≤ 0.05, ***P ≤ 0.001). Statistical tests were performed with Kruskal–Wallis test followed by Steel–Dwass–Critchlow–Fligner post hoc test.

We performed single mRNA tracking using 2MeSiR-labeled 2′-*O-*methyl RNA probes targeting poly(A)^+^ mRNA in COS7 cells with stress induced by applying 0.5 mM sodium arsenite for ≥60 min. By converting most of the probes into the dark state, we were able to visualize and track single mRNAs both inside and outside SGs in living cells (Supplementary Video 1). From acquired sequential images, we extracted trajectories lasting over 720 ms (40 frames) and analyzed the relationship between mean squared displacement (MSD) and time. Based on the MSD, we classified trajectories into three diffusion modes: stationary, confined and diffusive (Figure 2A, B) (35,36). The fractions of diffusion modes changed dramatically inside SGs when compared with the fractions in the cytoplasm outside SGs (Figure 2C). We found that the fraction of the stationary mode increased and became dominant inside SGs, whereas the fractions of confined and diffusive modes decreased. Furthermore, the confinement radius (*R*_conf_), diffusion coefficients for the confined mode (*D*_conf_) and diffusion coefficients for the diffusive mode (*D*_diff_) were calculated (Figure 2D–F; see Materials and methods for definitions). *D_diff_* of poly(A)^+^ mRNA was ∼0.17 μm^2^s^−1^ in the cytoplasm in unstressed cells, and ∼0.23 μm^2^s^−1^ in SGs in stressed cells. These values are in good agreement with the values of *D_diff_* of translating and untranslating mRNAs reported in a previous study (50). We detected diffusive mRNAs inside matured SGs, which showed little difference in MSD from cytoplasmic mRNAs (Figure 2B). For those diffusive mRNA molecules, there was no significant difference in *D*_diff_ between inside and outside SGs (*P*= 0.34, Kruskal–Wallis test followed by Steel–Dwass–Critchlow–Fligner post hoc test) (Figure 2F). These results suggest that mRNAs in SGs have heterogeneous behaviors with a large portion of mRNA anchored to some SG components and the remaining fractions moving diffusively in a similar manner to those outside SGs. These results are in good agreement with our previous studies using FRAP (14), in which we reported that ∼70% of the mRNA molecules showed immobile or slow diffusion and the remaining percentage moved freely. Supporting our results, Molliex *et al.* reported that the RNA recognition motifs and a low complexity sequence domain of RNA-binding proteins exist in two distinct types of environment in SGs (36). This heterogeneous organization of SGs would contribute to a wide variety of known and suggested cellular functions against stress, including translational arrest and degradation or translation reinitiation. Other notable findings include the difference in the dynamics of cytoplasmic (non-SGs) mRNAs between normal and stressed cells (Figure 2C–F). Our results showed that cytoplasmic mRNAs in stressed cells move faster than in normal cells (*P* < 0.05 and *P* < 0.001 for *D*_conf_ and *D*_diff_ respectively; Kruskal–Wallis test followed by Steel–Dwass–Critchlow–Fligner post hoc test). We hypothesized that polysome loss affects the change in *D_diff_* in the cytoplasm during stress. To examine this hypothesis, single mRNA tracking was performed in cells treated with emetine, a translation inhibitor, 60 min after the addition of sodium arsenite. We confirmed that emetine treatment prevented the formation of SGs upon stress (Supplementary Figure S2). The analysis of single mRNA tracking in stressed cells with emetine treatment showed that the ratio of the motility states of mRNAs in the cytoplasm (stationary/confined/diffusive) was similar to that in the absence of stress (Figure 2C). The quantitative dynamics parameters obtained from the single mRNA tracking showed that emetine treatment decreased *D_diff_* during stress (Figure 2D, E). In particular, *D_diff_* in the cytoplasm outside SGs showed a statistically significant decrease (***P < 0.001) compared to that without emetine (Figure 2F). This was also a statistically significant decrease (*P < 0.05) compared to *D_diff_* in SGs without emetine treatment (Figure 2F). These results indicate the effect of polysome loss as the cause of the change in *D_diff_* in the cytoplasm during stress.

### Super-resolution localization microscopy of poly(A)^+^ mRNA in SGs

**Figure 3.**
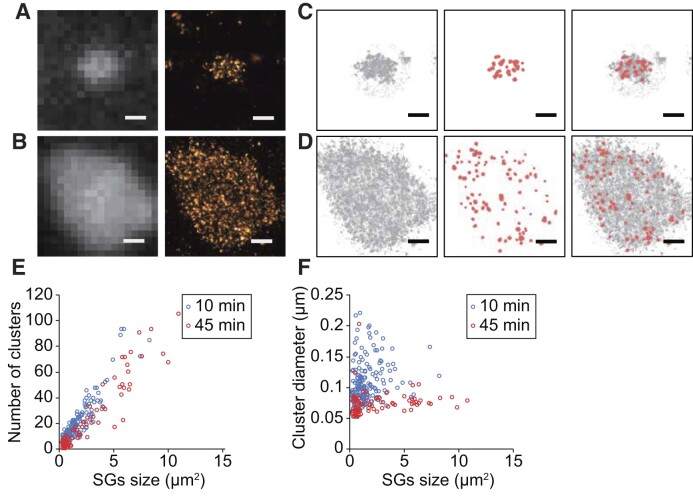
Super-resolution imaging of mRNA in SGs in fixed COS7 cells. (A, B) Exemplary images of poly(A)^+^ mRNA distributions around SGs acquired by epi-fluorescence microscopy (left) and super-resolution localization microscopy (right). COS7 cells were treated with 0.5 mM sodium arsenite for 10 min (**A**) and 45 min (**B**). Scale bars, 500 nm. (C, D) Exemplary of clusters detection with DBSCAN. Localization points shown in (A) and (B) were plotted after extraction of SGs regions (left). Core regions in SGs were automatically detected with DBSCAN algorithm (middle). Core regions were overlaid on the total localization points (right). Localization data shown in (A) and (B) were used in (**C**) and (**D**), respectively. Scale bars, 500 nm. (**E**) Relationship between the number of clusters and the SGs size. Red circles and blue hollow circles represent the data at 10 and 45 min after stress induction, respectively. (**F**) Relationship between the cluster diameters and the SGs size. Red hollow circles and blue hollow circles represent the data at 10 and 45 min after stress induction, respectively.

We performed super-resolution localization microscopy targeting poly(A)^+^ mRNAs inside SGs to identify the structural basis responsible for the dynamics of mRNAs in SGs. First, fixed samples were examined to confirm the fine distribution of mRNAs in SGs. After the addition of stress, cells were fixed by using aldehydes at time points of 10 or 45 min, representing the early and the late phases of SG formation, respectively. By analyzing the acquired nanoscale localization of mRNAs inside SGs, we confirmed that mRNAs localize heterogeneously in SGs and form several high-density clusters (cores) surrounded by low-density domains (shell regions), as reported previously (6) (Figure 3A). Comparison of two time points after the addition of arsenite (10 or 45 min) revealed that there is no obvious variation in the density of the cores. The number and size of cores detected were quantitatively analyzed by density-based spatial clustering of applications with noise (DBSCAN) (40). Here, we refer to the clusters extracted by DBSCAN with the following parameters as cores: epsilon with 25 nm and number of points with 100 points, where epsilon specifies the distance for considering two points as being in the same cluster and the number of points denotes the number of localized points. Under these conditions, each cluster is supposed to contain at least ten molecules within spatial resolution based on a rough estimation from the blinking analysis of Cy5 (data not shown). The quantitative analysis of these clusters revealed that the densities of cores were 10 cores/μm^2^ for both time points (∼11.7 cores/μm^2^ at 10 min and ∼8.82 cores/μm^2^ at 45 min, calculated as slopes of linear regression with zero intercept on scatter plots). Our results are comparable to the value of 4.7 cores/μm^3^ reported for G3BP in a previous study (6). The number of mRNAs per core quantified in our study is reasonable compared to the theoretical value (47 and see Supplementary Note 1). In addition, the size of each core does not increase but decreases despite the increased diameter of SGs (∼97 nm at 10 min and ∼71 nm at 45 min in diameter, median value) (Figure 3F). These results indicate that cores do not associate each other from early stage to late stage of SG maturation. By focusing on the gap areas between cores, we could confirm that SGs have a porous structure, indicating that there are some obstacles that mRNA molecules cannot go through.

### Simultaneous imaging of single mRNA dynamics and nanoscale localization of poly(A)^+^ mRNAs in SGs

**Figure 4.**
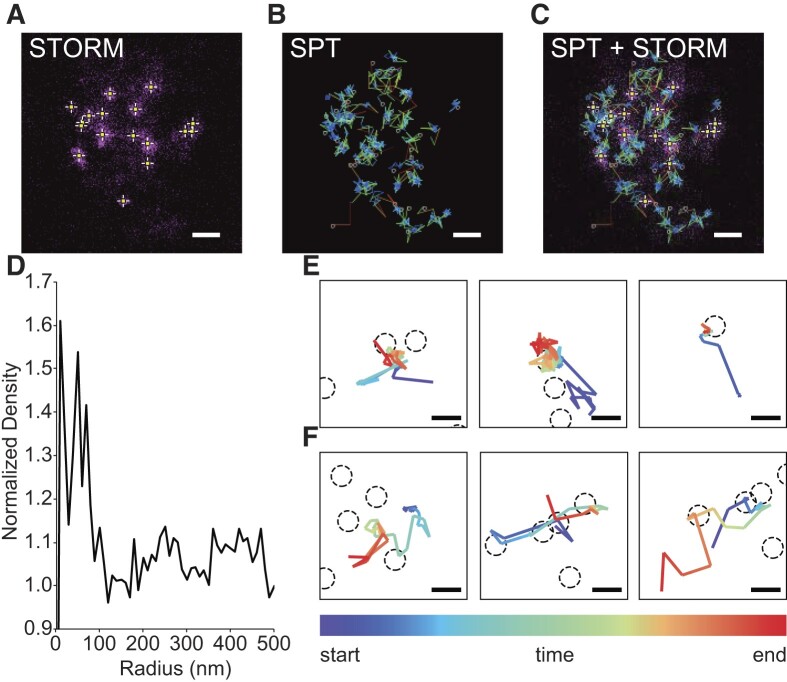
SPT-STORM analysis of mRNA in SGs in living COS7 cells. (A–C) Exemplary images of the combination analysis of super-resolution localization microscopy and single mRNA tracking in living COS7 cells. Poly(A)^+^ mRNA molecules were visualized by 2′-*O*-methyl RNA oligonucleotides labeled with HMSiR, for super-resolution microscopy, and Cy3B, for single mRNA tracking. Imaging was performed 60 min after the stress induction by 0.5 mM sodium arsenite. (**A**) A super-resolution image annotated with cores detected by DBSCAN, where yellow crosses show the centroids of cores. (**B**) Single mRNA trajectories of the same region shown in (A) color-coded based on the displacements between the two consecutive frames. (**C**) Merged image of (A) and (B). Scale bars, 500 nm. (**D**) Radial distribution function of tracked points. Radii were calculated as the distances between tracked points and its nearest cores centroids. Radial distributions were normalized with the data calculated with randomly generated points in exchange for the tracked points. (E, F) Exemplary trajectories of anchored (**E**) and drifting (**F**) molecules, color-coded based on the time from the start to the end. Scale bars, 200 nm.

We simultaneously performed single mRNA tracking and super-resolution localization microscopy in living cells to associate single mRNA dynamics with nanoscale localization. We simultaneously acquired two channels of images and analyzed each channel. In the combined results of tracking and localization, we observed that mRNA molecules are mainly found around cores. To quantitatively investigate the relationship between tracking points and cores, we calculated the radial distribution function (RDF) based on the distance between the detected points in tracks and their nearest cores. We found that the RDF showed high density in the short radius area (<100 nm), indicating that tracking points are frequently found around cores (Figure 4D). In addition, we examined the behavior of RNA molecules categorized into the diffusive mode around cores and found that some diffusive molecules slowed down around cores after several frames while others continued to freely diffuse (Figure 4E, F). These results suggest that cores consist of tethered mRNA molecules whose mobilities are restricted, which is consistent with the results showing that the stationary mode fraction increased in SGs when compared with the level of this fraction in the cytoplasm (Figure 2C).

### Single mRNA dynamics and nanoscale localization of poly(A)^+^ mRNAs during stress removal

**Figure 5.**
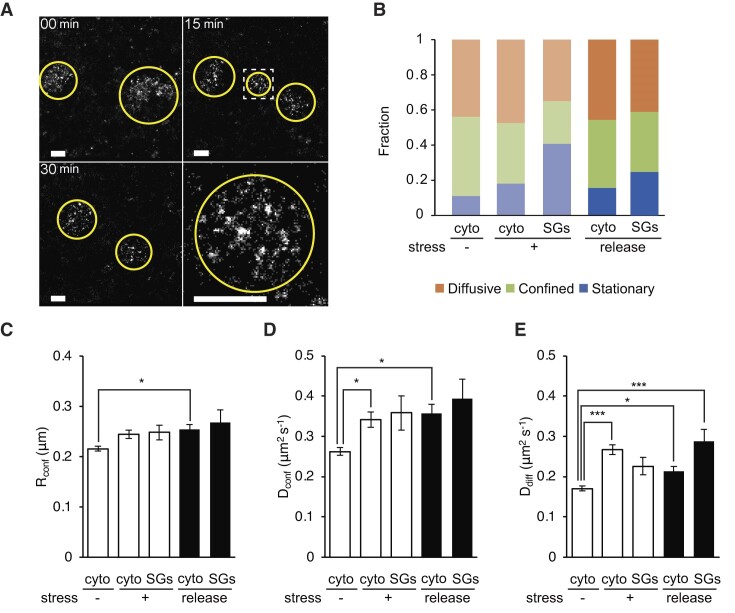
Behavior of mRNA in SGs in COS7 cells after releasing the stress. (**A**) Live super-resolution microscopy after stress release. Super-resolution images at 0, 15 and 30 min after removal of sodium arsenite are shown. The regions of SGs are indicated by yellow circles. An enlarged image of the dashed white region shown in the panel at the top right is presented in the panel at the bottom right. Scale bars, 1 μm. (**B**) Fraction of diffusive, confined, and stationary modes in the cytoplasm (cyto) in normal cells and in the cytoplasm or SGs in stressed and stress-relieved cells (mean ± s.e.m. for cells; *n* = 9 cells). (**C**) Radii of confinement calculated for each condition (mean ± s.e.m. for trajectories; *n* ≥ 58 trajectories from 9 cells; *P ≤ 0.05). (**D**) Diffusion coefficients for confinement mode calculated for each condition (mean ± s.e.m. for trajectories; *n* ≥ 58 trajectories from 9 cells; *P ≤ 0.05). (**E**) Diffusion coefficients for diffusion mode calculated for each condition (mean ± s.e.m. for trajectories; *n* ≥ 69 trajectories from 9 cells; *P ≤ 0.05, ***P ≤ 0.001). Statistical tests were performed with Kruskal–Wallis test followed by Steel–Dwass–Critchlow–Fligner post hoc test.

We performed nanoscale imaging of mRNA during SG dissociation upon removal of stress, an important process in understanding the function of SGs. Live super-resolution imaging results over 30 min after stress removal showed that the core retained its structure when SGs dissociated (Figure 5A and Supplementary Video 2). The core structure was maintained even during stress recovery, indicating that the core is an important component unit not only in SG formation but also SG dissociation. Based on single mRNA tracking analysis in SGs, it was found that the ratio of motility states changed, with the stationary mode decreasing and the confined and diffusive modes increasing, while the dynamics parameters showed little change when stress was removed (Figures 5B-E).

To confirm whether the leaving of single mRNA molecules can be observed during SGs dissociation, we comprehensively examined whether the starting and ending points of the detected tracks were inside or outside of SGs (Supplementary Figure S4). As a result, both influx (out→in) and efflux (in→out) were observed during stress (about 13% each), suggesting that the replacement of mRNA was occurring as expected from the FRAP results (14). In addition, the stationary mode in the fraction of mRNA that remained in SGs (in→in) decreased, and the fraction of mRNA transiting through SGs (out→out) increased during stress removal, indicating that the ability of SGs to retain mRNA was reduced (Supplementary Figure S4b, d). Furthermore, the efflux (in→out) increased while the influx (out→in) decreased considerably after the removal of stress, suggesting that the mechanisms regulating influx and efflux were different from those under stress. The results of observing mRNA efflux (in→out) from and transit through (out→out) SGs showed that mRNA exit from SGs can be detected by this comprehensive analysis. Although a small amount of mRNA efflux from SGs (in→out) was detected, it was intrinsically indistinguishable from the fraction of mRNA transiting through SGs (out→out) because of the difficulty in long-term tracking due to the short on-duration of the fluorophore used. Thus, it was not clear from our results that there was actually an efflux of mRNA that had stopped within the SGs.

## Discussion

In this study, we initially developed a technique to directly observe single mRNA dynamics inside SGs and quantified the ratio of the motility states of mRNA based on the diffusion coefficients (stationary: ∼40%, confined: ∼25%, freely diffusing: ∼35%), demonstrating that mRNA movements are suppressed inside SGs. Next, super-resolution localization microscopy showed that mRNAs inside SGs distribute heterogeneously forming high-density cores with diameters of ∼97 nm in early small SGs (10 min after stress induction) and ∼71 nm in late large SGs (45 min) and a density of ∼11.7 cores/μm^2^ in early small SGs and ∼8.82 cores/μm^2^ in late large SGs. Subsequently, by combining single mRNA tracking and super-resolution localization microscopy we observed that most mRNAs are anchored to cores while some mRNA diffuse around cores. Finally, the observation of the localization and dynamics of mRNA during stress removal suggested that mRNA remains regulated by the core even after stress removal. These results suggest that the highly-organized structure of SGs serves to provide a platform for mRNA association. The dynamic properties of mRNA inside SGs (i.e. transient interaction with cores) support the hypothesis that the components of cores in SGs show liquid droplet behavior. This diverse mRNA dynamics and environment in SGs may be responsible for quick and reversible responses to cellular stresses.

Compared with other mRNA imaging techniques (16–23), our approach has the following advantages. First, owing to the blinking characteristics of fluorophores attached to probes, we can visualize single mRNA molecules even if they are placed in a densely-packed environment, which is advantageous when analyzing highly expressed mRNA. In labelling methods based on signal amplification using multiple binding sites (18,23), it would be difficult to resolve single molecules in dense regions because signals from multiple target molecules placed within spatial resolution would disturb each other. The use of other conventional methods (19–22) that use non-blinking fluorophores for observing mRNA in granular structures would require the adjustment of the labelling ratio, which is tedious work and would also result in a low number of detections because of the reduced labelling ratio. Second, the design of probes is simple because our strategy is based on hybridization of ∼20 base-oligonucleotides without genetic engineering. This simple and flexible approach can label mRNAs in a sequence-specific manner, as reported previously (14,22). Our genetic engineering-free method enables the analysis of intact endogenous mRNA without any sequence modifications. Third, we have a variety of fluorophore choices for attachment to mRNA. In general, synthetic fluorophores show brighter fluorescence and better photostability with smaller molecular weights than fluorescent proteins. In this study, we quantitatively evaluated three types of fluorophores that have distinct blinking characteristics depending on their basic structures. The variety of blinking properties facilitated the use of different dyes for each investigation (i.e. single particle tracking and super resolution imaging). Massive screening of fluorophores for blinking-based tracking must be assessed for future development of this method.

We demonstrated that a combination of super-resolution imaging and single mRNA tracking can be used for investigating cellular structures based on molecular dynamics and vice versa. This technique can be applied to investigate other cellular structures involved in mRNA metabolism, such as the nuclear speckles (40), the cytoskeleton (41,42) and the nuclear pore complex (43).

Recently, phase separation of intracellular macromolecules, including proteins and nucleic acids, has attracted the attention of researchers because of its ability to organize non-membrane bound organelles (44). Several studies indicate that LLPS triggered by intrinsically disordered regions (IDRs) of SG components plays significant roles in the formation of SGs (6,11,45,48). However, how and when LLPS is involved in the process of formation of SGs remains elusive. We analyzed the dynamics of mRNAs in SGs at the single molecule level in living cells and revealed that they show a wide variety of mobilities including stationary, confined and diffusive. We also demonstrated that more than half of the mRNA molecules are dynamically moving, i.e. confined and diffusive, at the single molecule level in mature SGs. These results indicate that SGs are not only random aggregates but well-organized structures that involve various states of mRNA molecules. The diffusion and transient interaction of mRNA in core structures indicate characteristics of LLPS. Our approach and findings contribute to understanding the behavior of RNAs in structures that LLPS is involved in.

Recent studies using super-resolution fluorescence microscopy clarified that SGs consist of cores and shell regions (6,8–11). However, the detailed characteristics of cores, especially their relevance to mRNA, which is a key molecule in SGs, have been little investigated (16,17). In this study, we observed the dynamics of mRNA molecules in SGs at the single molecule level and their association with cores. Cores were found to function by tethering mRNA molecules, thereby confirming that cores function as structural units in SGs. The mechanisms on how this tethering function is organized represent an interesting problem to solve in the future. Comparing mRNA dynamics and localization at the nanometer scale in various types of RNA granules *in vitro* and *in vivo* would provide clues on this problem. In particular, investigating the behaviors of mRNA in P bodies is a worthy further study because they locate close to SGs in mammalian cells (15). Understanding the possible interaction of mRNA molecules between SGs and P bodies would further reveal the function and outcome of mRNAs in stressed cells (16,17).

Although this single mRNA tracking and analysis method was able to observe mRNA exit, the proportion of mRNA that effluxed and passed through SGs during stress removal did not increase relative to the proportion during stress. This result suggests that mRNA trapped in SGs cannot escape even after stress recovery and that mRNA does not immediately return to its unstressed state (i.e. translatable state bound to the ribosome). Here we considered two possible hypotheses for this reason; first, the core retention is reduced during the dissociation process of SGs, and the mRNAs in SGs return to a steady state in a confined mode. In this mechanism, mRNAs recruited into SGs do not emerge into the cytoplasm in a diffusive state, and therefore, they may not be seen exiting the SGs. This hypothesis is reasonable considering that LLPS formation is accompanied by interactions through IDRs and transitions to a stable state by RNA aggregation. It is possible that over several hours after the removal of stress, the RNA-binding proteins are regulated by changes in their configuration. These processes are important in considering the reversibility of SG formation. The second hypothesis is that mRNAs incorporated into SGs are eventually degraded and fragmented into small pieces. In this case, the speed of diffusion would be too fast to track. The number of mRNAs in SGs is only about 10% of the total intracellular mRNAs, and the majority resides outside of SGs. The fact that translation function is restored within a few hours after the removal of stress (16) may be due to the change of mRNAs outside SGs to a translatable state. Considering the stress-activated ribosome-associated quality control (saRQC) found in a recent study (46), mRNAs incorporated into SGs may enter a special degradation pathway and not exit from SGs. Observation of this phenomenon is difficult with our method because of the limited trackable time. We expect to understand more in the future by refining our method to be able to track molecules for longer durations.

Our results indicate that cores in SGs are not static but dynamic structures that form by recruiting mRNA molecules diffusing in the near vicinity. Taking into consideration the results obtained in FRAP experiments (14,15), mRNA molecules exchange between SGs and the cytoplasm. Taken together, we found that mRNA molecules change the mobility inside SGs showing increase in stationary mode fraction, whereas part of mRNA can still freely diffuse. The construction of large granules by assembling small cores that can reversibly form and disassemble represents an effective way for fast adaptation to environmental changes under stress.

## Supplementary Material

gkae588_Supplemental_Files

## Data Availability

All data are available in the manuscript or in the supplementary information. Materials that support the findings of this study are available from the corresponding author upon request.
