# Supplementary material for: Nanoscale dynamics and localization of single endogenous mRNAs in stress granules
Source: Nucleic Acids Res. 2024 Jul 29;52(15):8675–86. doi: 10.1093/nar/gkae588 (PMC11347133; doi:10.1093/nar/gkae588)
Supplement: gkae588_Supplemental_Files [file gkae588_supplemental_files.zip › Sugawara et al_SupplementaryInformation_revised3.pdf]

Supplementary Information for
Nanoscale dynamics and localization of single endogenous mRNAs in stress granules

Ko Sugawara, Shin-nosuke Uno, Mako Kamiya, Akihiko Sakamoto, Yasuteru Urano, Takashi Funatsu, and Kohki Okabe

Supplementary Note 1 | Estimation of the number of mRNAs per core in SGs.

The number of mRNAs per cell is estimated to be around 100,000-1,000,000 (Miro et al., 2015). Based on the report that about 10% of these are recruited to SGs (Khong et al., 2017) and the number of SGs at 60 min of stress load (Zhang et al., 2011) is about 20, the estimated number of mRNA molecules per SG is about 5,000. Since the observed densities of cores were 10 cores/μm^2 and the size of SGs at 60 minutes of stress exposure averages 3 μm^2 (Zhang et al., 2011), there are about 30 cores per SG, and the number of mRNAs per core is estimated to be about 10-100 molecules, taking into account that some mRNAs are distributed in the shell.

Supplementary Table 1 | Summary of blinking properties of Cy5, ATTO647N and 2MeSiR.
Number of blinking events, fluorescence-on duration per event, fluorescence-off duration per event and number of photons per frame were measured for Cy5 (n = 17 particles), ATTO647N (n = 68 particles) and 2MeSiR (n = 75 particles) in the PBS (pH 7.4)-based imaging buffer containing 14.3 mM βME. Mean values are represented in the table. For Cy5 and ATTO647N, the fluorescence-off duration was not obtained because the number of blinking events was insufficient.

Dye	Blinking cycles	On duration (s)	Off duration (s)	Photons per frame
Cy5	1.0	0.40	NA	253
ATTO647N	1.2	10	NA	506
2MeSiR	14	1.0	2.2	268

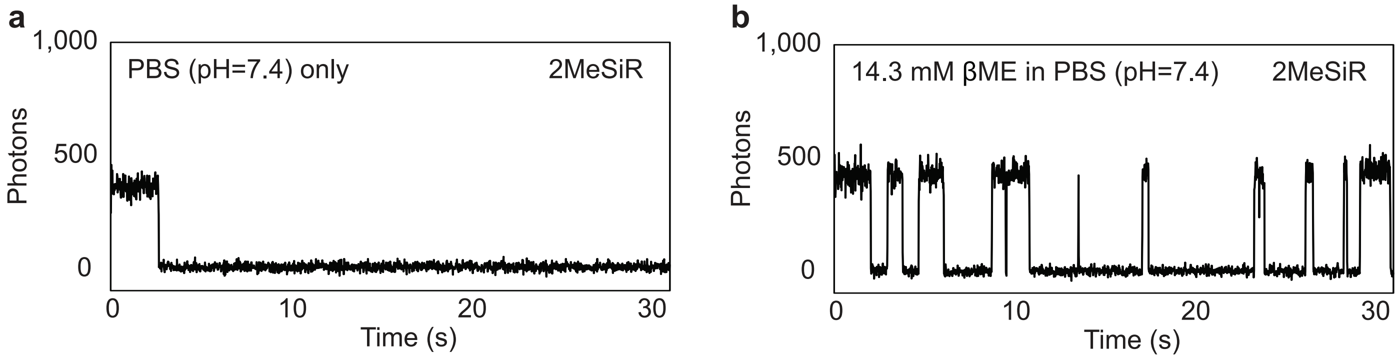

Supplementary Figure 1 | The influence of thiol on the blinking of 2MeSiR.
(**a**, **b**) Exemplary single-molecule fluorescence time traces for 2MeSiR in the absence (**a**) and presence (**b**, **Figure 1d**) of βME. Poly(U)$_{22}$ 2′-*O*-methyl RNA-bound fluorophores were attached to the coverslip.

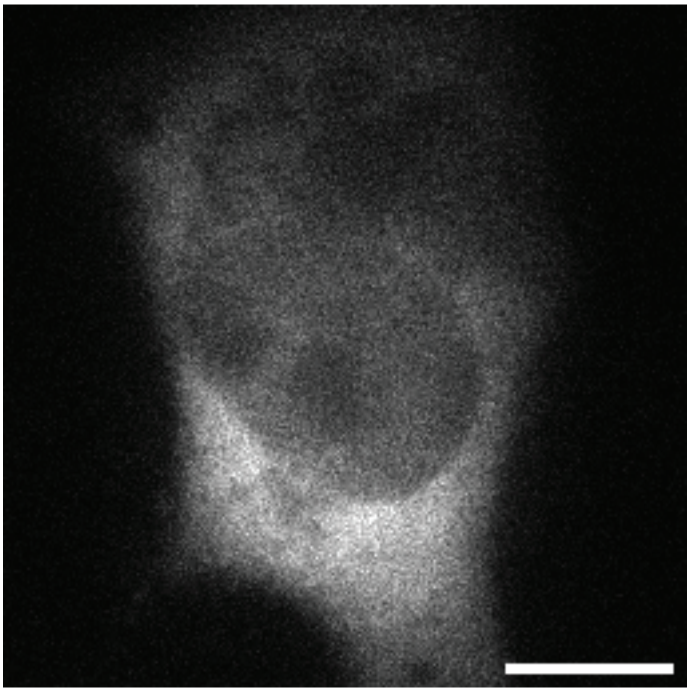

Supplementary Figure 2 | Poly(A)$^+$ mRNA imaging in a stressed COS7 cell with emetine treatment. Exemplary epi-fluorescence imaging of poly(A)$^+$ mRNA in a stressed COS7 with emetine treatment. SGs were not observed. Scale bar, 10 µm.

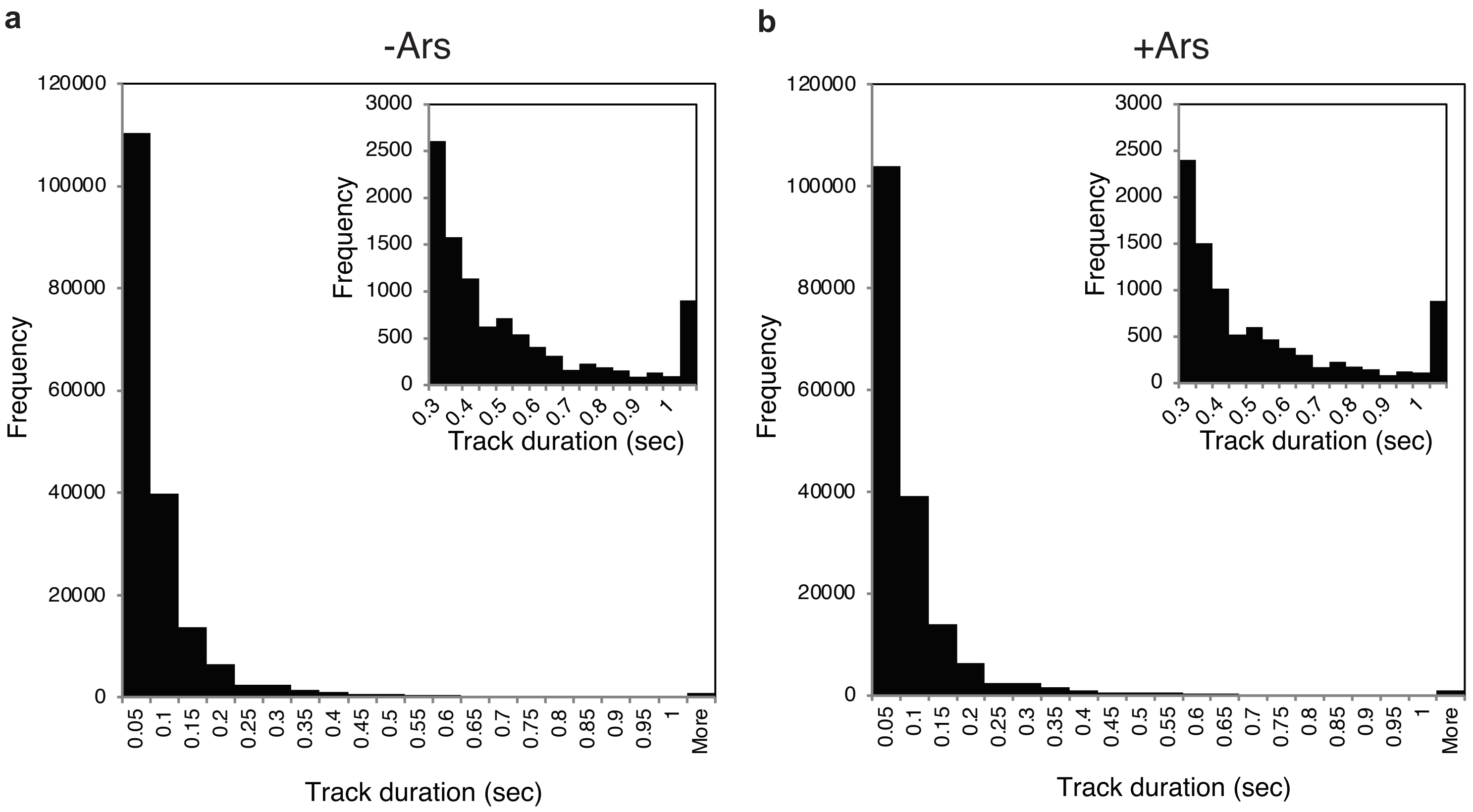

Supplementary Figure 3 | Distribution of track durations of 2MeSiR-labeld poly(U)$_{22}$ 2′-*O*-methyl RNA in COS7 cells.

(**a**, **b**) Histograms of the distribution of track durations of 2MeSiR-labeled poly(U)$_{22}$ 2′-*O*-methyl RNA in normal cells (**a**) and in stressed cells (**b**). Track durations above 0.3 seconds were displayed in the inset at the top right.

a

	Under stress			
	in → in	in → out	out → in	out → out
Stationary	109	2	3	13
Confined	30	16	15	15
Diffusive	30	22	24	33
Sum	169	40	42	61

b

	Under stress			
	in → in	in → out	out → in	out → out
Stationary	0.349	0.006	0.010	0.042
Confined	0.096	0.051	0.048	0.048
Diffusive	0.096	0.071	0.077	0.106
Sum	0.542	0.128	0.135	0.196

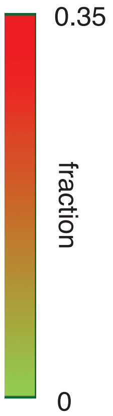

c

	Recovery from stress			
	in → in	in → out	out → in	out → out
stationary	35	2	3	2
confined	25	6	1	19
diffusive	15	17	0	27
sum	75	25	4	48

d

	Recovery from stress			
	in → in	in → out	out → in	out → out
stationary	0.230	0.013	0.020	0.013
confined	0.164	0.039	0.007	0.125
diffusive	0.099	0.112	0.000	0.178
sum	0.493	0.164	0.026	0.316

Supplementary Figure 4 | Influx and outflux of mRNA under stress and after releasing the stress.
(**a**, **b**) Tables illustrating mRNA influx and outflux in stressed COS7 cells. Columns were divided based on whether the tracking start and end points were inside or outside of SGs, and rows were divided based on the diffusion mode. The absolute numbers of tracks were summarized in (**a**), and the ratios were summarized in (**b**) (collected from 9 cells). (**c**, **d**) The same for (**a**) and (**b**), but in COS7 cells after releasing the stress (collected from 9 cells). In tables (**b**) and (**d**), color-coding was applied using a continuous scale where 0 is represented in green and 0.35 in red.

Supplementary Video 1 | Single endogenous poly(A)$^+$ mRNA imaging in a stressed COS7 cell.
Real-time fluorescence imaging of poly(A)$^+$ mRNA in a stressed COS7 cell. The movie was acquired at 55 Hz, in which the yellow broken lines indicate the positions of SGs. Scale bar, 5 μm.

Supplementary Video 2 | Live super-resolution microscopy of poly(A)$^+$ mRNA after stress release.
Video of super-resolution imaging of poly(A)$^+$ mRNA in a COS7 cell after removal of sodium arsenite. The caption in the upper left indicates the elapsed time after stress removal.
